# Hypertensive Anaphylaxis in the Emergency Department: A Case Series

**DOI:** 10.7759/cureus.103811

**Published:** 2026-02-17

**Authors:** Pramod Debnath, Aakash Verma, K Anup Kumar

**Affiliations:** 1 Emergency Medicine, Command Hospital Air Force, Bangalore, IND

**Keywords:** allergic reaction, emergency medicine and trauma, fluid resuscitation, hypertensive anaphylaxis, life threatening anaphylaxis

## Abstract

Anaphylaxis is a rapid-onset, life-threatening allergic reaction that classically presents with hypotension and distributive shock. Hypertension is generally considered atypical in this context and may delay recognition of anaphylaxis in the emergency department. However, case reports and small studies have described paradoxical “hypertensive anaphylaxis,” with reported prevalence rates of up to 12.9%. Awareness of this variant is essential to avoid withholding timely epinephrine therapy.

We describe four adults who presented to the emergency department with systemic allergic reactions characterized by urticaria, angioedema, dyspnea, and gastrointestinal involvement. All four patients were initially normotensive but subsequently developed paradoxical hypertension, with blood pressures ranging from 140/90 mmHg to 168/102 mmHg at the time of anaphylaxis. Triggers included intravenous fluids, medications, and an insect bite. Each patient received intramuscular epinephrine (0.5 mg), intravenous fluids, corticosteroids, antihistamines, and inhaled bronchodilators where appropriate. Two patients required repeat epinephrine dosing for persistent symptoms. Despite presenting with elevated blood pressure, all patients tolerated epinephrine without any complications related to the catecholamine surge, such as arrhythmia, myocardial ischemia, or intracranial events. All recovered fully and were discharged after a short period of hospital observation.

This case series reinforces that hypertension does not preclude the diagnosis of anaphylaxis and should not deter clinicians from administering first-line epinephrine. The pathophysiology of hypertensive anaphylaxis is not fully understood but may involve compensatory sympathetic activation, endogenous catecholamine release, or anxiety-mediated responses. Recognition of this variant is critical for emergency physicians, as reliance on hypotension as a diagnostic criterion can delay life-saving treatment.

In conclusion, hypertensive anaphylaxis represents an under-recognized clinical presentation. Our four cases add to the growing body of literature confirming that intramuscular epinephrine is safe and effective, even in the setting of elevated blood pressure. Emergency clinicians should base the diagnosis of anaphylaxis on systemic features rather than hemodynamic profile, ensuring that adrenaline administration is not delayed by paradoxical hypertension.

## Introduction

Anaphylaxis is a rapidly progressive, life‑threatening systemic hypersensitivity reaction that involves skin/mucosa, respiratory, cardiovascular, and/or gastrointestinal systems after exposure to a trigger; diagnosis is clinical and does not require hypotension per se [1-3]. Epidemiologic data suggest a global incidence of approximately 50-112 episodes per 100,000 person‑years and a lifetime prevalence of around 0.3%-5.1%, with medications and foods as leading triggers in adults and children, respectively [4]. Although distributive shock with hypotension is classically expected, a paradoxical hypertensive phenotype of anaphylaxis is increasingly recognized and may be underdiagnosed in emergency settings [1,2,3,5-7]. In a retrospective cohort examining ED presentations, hypertension at onset was observed in roughly 12.9% of anaphylaxis cases, cautioning against over‑reliance on hypotension for diagnosis [5]. Proposed mechanisms for hypertensive anaphylaxis include an early sympathetic/catecholaminergic surge, anxiety‑mediated adrenergic activation, and mediator‑driven increases in cardiac output; these are not mutually exclusive [1,8]. Across international guidelines, intramuscular adrenaline is emphasized as the first‑line, time‑critical therapy for anaphylaxis-even when the initial blood pressure is elevated-while antihistamines and corticosteroids remain adjunctive [1,2,3,9]. Case reports of hypertensive anaphylaxis further suggest that appropriately dosed intramuscular adrenaline is well-tolerated without hypertensive crises or ischemic complications [6,7]. Against this backdrop, we present a four‑patient ED case series demonstrating paradoxical hypertension at onset, rapid response to guideline‑concordant therapy [10], and favorable outcomes.

## Case presentation

Case 1

A 37-year-old, previously healthy woman presented to the Emergency Department (ED) on 26 April 2024 at 09:00 hours with complaints of one episode of non-bilious vomiting and two episodes of loose, watery stools over the previous 24 hours. She had no history of allergies, chronic illness, or regular medication use. On arrival, she was conscious, oriented, and mildly dehydrated. Baseline vital signs were: blood pressure (BP) 132/78 mmHg, heart rate (HR) 96 bpm, respiratory rate (RR) 16 breaths/min, temperature (Temp) 98.6 °F, and oxygen saturation (SpO)₂ 98% on room air. The patient was triaged as Emergency Severity Index (ESI) 3 and diagnosed with acute gastroenteritis. She received IV pantoprazole 40 mg, IV ondansetron 8 mg, and 500 mL of Ringer’s lactate over 2 hours. At 14:20 hours, she developed lip swelling, generalized urticaria, dyspnea, and bilateral wheeze. Vitals now showed BP 158/96 mm Hg, HR 128 bpm, RR 34/min, and SpO₂ 98%. She was re-triaged as ESI 1 and diagnosed with anaphylaxis with paradoxical hypertension. She received IM adrenaline 0.5 mg (1:1000), IV normal saline (0.9% NaCl) bolus, IV chlorpheniramine 47.5 mg, IV hydrocortisone 200 mg, and nebulized bronchodilator (levosalbutamol + ıpratropıum bromıde). As symptoms persisted, a second IM adrenaline 0.5 mg was administered. Within 5 minutes, symptoms resolved and BP normalized to 110/68 mm Hg, HR 96 bpm, RR 22 /min, and SpO2 98%. She was observed for 12 hours and discharged home after an uneventful recovery.

Case 2

A 32-year-old woman presented on 3 May 2024 with para-umbilical abdominal pain and loose stools for one day. She had no history of allergies or chronic medical illness. On examination, she appeared dehydrated but was alert. Initial vitals were: BP 114/70 mm Hg, HR 104 bpm, SpO₂ 97%, Temp 98.2 °F. She was triaged ESI 4 and diagnosed as acute enteritis. She received IM dicyclomine 10 mg and IV Ringer’s lactate. After approximately 200 mL of RL, she developed acute epigastric pain, vomiting, generalized urticaria, and dyspnea with bilateral wheeze. Repeat vitals showed BP 164/98 mm Hg, HR 124 bpm, RR 28/min, and SpO₂ 97%. She was re-triaged ESI 1 and diagnosed with anaphylaxis likely triggered by infusion fluid. She received IM adrenaline 0.5 mg repeated twice at 5-minute intervals, IV normal saline (0.9% NaCl) 1000 mL, IV chlorpheniramine 47.5 mg, IV hydrocortisone 200 mg, and inhaled bronchodilator (levosalbutamol + ıpratropıum bromıde). Symptoms improved within 15 minutes. BP returned to 108/64 mm Hg, HR 90 bpm, RR 20 /min, and SpO2 96%. She was admitted for observation and discharged uneventfully after 24 hours.

Case 3

A 24-year-old woman with no comorbidities presented on 31 July 2024 at 10:30 hours with fever (maximum 101 °F), chills, and nausea for one day. She had no known allergies. On arrival, she was alert and mildly dehydrated. Baseline vitals: BP 110/70 mm Hg, HR 96 bpm, SpO₂ 98%. She was triaged ESI 4 and treated with oral antipyretics and IV normal saline (0.9% NaCl) 500 mL. At 12:08 hours, she developed facial and lip swelling and generalized urticaria. Vitals were: BP 168/102 mm Hg, HR 134 bpm, RR 24/min, and Temp 97.8 °F. She was re-triaged ESI 1 and diagnosed with anaphylaxis with paradoxical hypertension. She received two doses of IM adrenaline 0.5 mg (5 minutes apart), IV Ringer’s lactate 1 L bolus, IV chlorpheniramine 47.5 mg, and IV hydrocortisone 200 mg. Ten minutes later, symptoms resolved, BP normalized to 114/68 mm Hg, HR 88, RR 20, and SpO2 97%, and she was admitted for observation for one day and discharged without complications.

Case 4

A 29-year-old male with a history of chronic urticaria presented on 9 May 2025 at 08:00 hours after an insect bite to the left little finger. He had localized pain and erythema. Initial vitals were: BP 140/90 mm Hg, HR 79 bpm, RR 22/min, SpO₂ 98%. He was triaged ESI 3 and given IM chlorpheniramine. At 08:10 hours, he developed a dry cough, hoarseness of voice, vomiting, and generalized urticaria. Vitals were: BP 152/95 mm Hg, HR 95 bpm, RR 24/min, and SpO₂ 96%. He was re-triaged ESI 1 and diagnosed with anaphylaxis secondary to an insect bite. He received IM adrenaline 0.5 mg (single dose), IV Ringer’s lactate 1 L, IV hydrocortisone 200 mg, and IV ondansetron 8 mg. Within 5 minutes, symptoms resolved and vitals normalized (BP 128/84 mm Hg; HR 78 bpm; RR 18/min; SpO₂ 98%). He was observed for several hours and discharged after an uneventful recovery.

Table 1 presents the vital signs and routine laboratory findings for all four cases.

**Table 1:** Vital signs and routine laboratory findings for the four patients BP = blood pressure; HR = heart rate; RR = respiratory rate; SpO₂ = peripheral capillary oxygen saturation; Hb = hemoglobin; WBC = white blood cells; Plt = platelets; IM = intramuscular

Parameter (Unit)	Case 1	Case 2	Case 3	Case 4	Normal ranges
Initial (pre-anaphylaxis)					
Blood Pressure (mm Hg)	132/78	114/70	110/70	140/90	90/50 - 135/85
Heart Rate (bpm)	96	104	96	79	60-100
Respiratory Rate (breaths/min)	16	18	18	22	12-20
SpO₂ (%)	98	97	98	98	>94
Temperature (°F) - axillary	98.6	98.2	97.6	98.9	97-100
At the onset of anaphylaxis					
Peak Blood Pressure (mm Hg)	158/96	164/98	168/102	152/95	90/50 - 135/85
Heart Rate (bpm)	128	124	134	95	60-100
Respiratory Rate (breaths/min)	34	28	24	24	12-20
SpO₂ (%)	98	97	96	96	>94
Temperature (°F) - axillary	98.6	97.8	97.8	98.4	97-100
Post-treatment (after IM ADRENALINE)					
Blood Pressure (mm Hg)	110/68	108/64	114/68	128/84	90/50 - 135/85
Heart Rate (bpm)	96	90	88	78	60-100
Respiratory Rate (breaths/min)	22	20	20	18	12-20
SpO₂ (%)	98	96	97	98	>94

## Discussion

Anaphylaxis is classically recognised by cardiovascular collapse (hypotension), distributive shock, and airway compromise [1,2]. Hypertension at presentation is therefore counterintuitive and often delays recognition. Our series of four patients illustrates that paradoxical hypertension is not only possible but may be more common than previously appreciated, with a reported prevalence of up to 12.9% in one retrospective cohort [5]. Several mechanisms have been proposed. One is a compensatory sympathetic surge mediated by endogenous catecholamine release in response to early anaphylactic vasodilation [9]. Another hypothesis is that mast cell-derived mediators, such as histamine, acting on H1 and H2 receptors to produce tachycardia and increased cardiac output, temporarily elevate blood pressure [9]. Exogenous triggers, anxiety, and baseline autonomic tone may further accentuate this paradoxical response. Importantly, these pathophysiologic explanations are not mutually exclusive [9]. Our patients all presented with typical mucocutaneous and respiratory features of anaphylaxis-urticaria, angioedema, and wheeze, yet had blood pressures consistently above 140/90 mmHg. The key clinical point is that elevated blood pressure should not exclude the diagnosis of anaphylaxis. In fact, reliance on hypotension as a diagnostic criterion risks delayed recognition and treatment, with potentially fatal consequences [10]. A practical concern in such cases is whether intramuscular adrenaline is safe when baseline blood pressure is elevated. All four of our patients received standard dosing (0.5 mg IM), and two required repeat doses. None developed hypertensive crisis, arrhythmia, or ischemic complications. This supports prior isolated reports that adrenaline remains both safe and effective even in hypertensive anaphylaxis [3,4,6-8]. Clinicians should resist the temptation to withhold adrenaline out of concern for exacerbating blood pressure.

## Conclusions

Hypertension during anaphylaxis is an uncommon but clinically important variant that can obscure recognition in the emergency department. Our four cases highlight that blood pressure alone should not be used to rule out anaphylaxis when characteristic mucocutaneous, respiratory, or gastrointestinal features are present. Prompt intramuscular adrenaline was safe and effective in all patients, despite initial hypertension. Emergency physicians should maintain a high index of suspicion for anaphylaxis regardless of hemodynamic profile, and treatment decisions should prioritize rapid adrenaline administration over concerns about transient blood pressure elevations.
